# A Modified Multiplicative Thinning-Based INARCH Model: Properties, Saddlepoint Maximum Likelihood Estimation, and Application

**DOI:** 10.3390/e25020207

**Published:** 2023-01-21

**Authors:** Yue Xu, Qi Li, Fukang Zhu

**Affiliations:** 1 School of Mathematics, Jilin University, Changchun 130012, China; 2 College of Mathematics, Changchun Normal University, Changchun 130032, China

**Keywords:** INARCH model, saddlepoint approximation, thinning-based model, time series of counts

## Abstract

In this article, we propose a modified multiplicative thinning-based integer-valued autoregressive conditional heteroscedasticity model and use the saddlepoint maximum likelihood estimation (SPMLE) method to estimate parameters. A simulation study is given to show a better performance of the SPMLE. The application of the real data, which is concerned with the number of tick changes by the minute of the euro to the British pound exchange rate, shows the superiority of our modified model and the SPMLE.

## 1. Introduction

In practice, we can often observe a series of integer-valued data that have their own distinguishing characteristics, and many models were proposed for modeling integer-valued time series, such as the integer-valued autoregressive (INAR) process introduced by McKenzie (1985) [1], and Al-Osh and Alzaid (1987) [2]; the integer-valued moving average process proposed by Al-Osh and Alzaid (1988) [3]; the integer-valued autoregressive moving-average model defined by McKenize (1988) [4]; and the integer-valued generalized autoregressive conditional heteroscedasticity (INGARCH) model proposed by Ferland et al. (2006) [5], among others. Here we focus on two kinds of the models above: one is the INAR process, which was introduced as a convenient way to transfer the usual autoregressive structure to a discrete-valued time series, and a *p*-order model, which is defined as follows:

$$X_{t}=\sum_{i=1}^{p}\alpha_{i}\circ X_{t-i}+\varepsilon_{t},$$

where $\alpha_{i}\in \left[0,1\right)$ for i=1,…,p, and $\left\{\varepsilon_{t}\right\}$ is a sequence of independent and identically distributed (i.i.d.) non-negative integer-valued random variables with $E(\varepsilon_{t})=\mu$ and $\mathrm{Var}\left(\varepsilon_{t}\right)=\sigma_{\varepsilon }^{2}$. The binomial thinning operator ∘ is defined by Steutel and Van Harn (1979) [6] as:

$$\alpha \circ X=\sum_{i=1}^{X}Y_{i},\;\mathrm{if}\;X>0\;\mathrm{and}\;0\;\mathrm{otherwise},$$

where $Y_{i}$ are i.i.d. Bernoulli random variables, independent of *X*, with a success probability are defined by α. This model has been generalized by Qian and Zhu (2022) [7], and Huang et al. (2023) [8], among others.

The other is the INGARCH model which was proposed by Ferland et al. (2006) [5] to model the observations of integer-valued time series which exist heteroscedasticity; this INGARCH(p,q) model with a Poisson deviate is defined as:

$$X_{t}|F_{t-1}:P\left(\lambda_{t}\right),\;\;\;\lambda_{t}=\alpha_{0}+\sum_{i=1}^{p}\alpha_{i}X_{t-i}+\sum_{j-1}^{q}\beta_{j}\lambda_{t-j},$$

where $\alpha_{0}>0,\alpha_{i}\geq 0,\beta_{j}\geq 0,i=1,\ldots ,p,j=1,\ldots ,q,p\geq 1,q\geq 0$, and $F_{t-1}$ is the σ-field generated by $\left\{X_{t-1},X_{t-2},\ldots \right\}$. This model has been generalized by Hu (2016) [9], Liu et al. (2022) [10], and Weiß et al. (2022) [11], among others. Weiß (2018) [12] and Davis et al. (2021) [13] gave recent reviews. According to definitions of INAR and INGARCH models, we noticed that the INAR model is thinning-based, while the INGARCH model is specified by a conditional distribution with a time-varying mean depending on past observations. Combining the thinning-based stochastic equations and the INGARCH model, Aknouche and Scotto (2022) [14] proposed a multiplicative thinning-based INGARCH (MthINGARCH) model to model the integer-valued time series with high overdispersion and persistence. Furthermore, it fits well with heavy-tailed data regardless of the choice of innovation distribution and does not require recourse to complex random coefficient equations. The MthINGARCH model is denoted by:

(1) $$\left\{\begin{array}{c} X_{t}=\lambda_{t}\varepsilon_{t},\\ \lambda_{t}=1+\omega \circ m+\sum_{i=1}^{q}\alpha_{i}\circ X_{t-i}+\sum_{j=1}^{p}\beta_{j}\circ \lambda_{t-j}, \end{array}\right.$$

where the symbol ∘ stands for the binomial thinning operator, and 0≤ω≤1, $0\leq \alpha_{i}<1$ and $0\leq \beta_{j}<1\;\left(i=1,\ldots ,q,j=1,\ldots ,p\right)$, *m* is a fixed positive integer number that was introduced for more flexibility. Since there is no explicit probability mass function for the series $\left\{X_{t}\right\}$, then the traditional maximum likelihood estimation (MLE) cannot be applied to estimate the parameters; therefore, Aknouche and Scotto (2022) [14] used a two-stage weighted least squares estimation instead.

Note that the probability mass function of the random variables cannot be given directly for the likelihood function in some cases; to solve this problem, saddlepoint approximation has been proposed. Daniel (1954) [15] introduced saddlepoint techniques into the statistical field, which have been extended by Field and Ronchetti (1990) [16], Jensen (1995) [17], and Butler (2007) [18]. Saddlepoint techniques have been used successfully in many applications because of the high accuracy with which they can approximate intractable densities and tail probabilities. Pedeli et al. (2015) [19] proposed an alternative approach based on the saddlepoint approximation to log-likelihood, and the saddlepoint maximum likelihood estimation (SPMLE) was used to estimate the parameters of the INAR model, which demonstrates the usefulness of this technique. Thus, through combining the MthINGARCH model of Aknouche and Scotto (2022) [14] and the saddlepoint approximation, we propose a modified multiplicative thinning-based INARCH model for modeling high overdispersion, before applying the saddlepoint method to the estimated parameters. Although the two-stage weighted least squares estimation could be used to estimate the parameters of our modified model, we still adopted the SPMLE as it was still expected to have a better performance than the two-stage weighted least squares estimation in practice. Here, we just consider the INARCH model instead of the INGARCH model because it is difficult and complex to give the conditional cumulant-generating function of random variables for the latter model when applying the saddlepoint approximation.

This article has the following structure. A modified multiplicative thinning-based INARCH model is given, alongside some related properties in Section 2. Moreover, we use the Poisson distribution and geometric distribution for innovations. Section 3 discusses the SPMLE and its asymptotic properties, then simulation studies for both models with SPMLE are also given. A real data example is analyzed with our modified models in Section 4, and comparisons with existing models are made. In-sample and out-of-sample forecasts are used to show the superiority of the SPMLE and our modified model. The conclusion is given in Section 5. Some details of SPMLE and proof of some theorems are presented in the Appendix A.

## 2. A Multiplicative Thinning-Based INARCH Model

Note that N={0,1,2,…} and Z={…,−1,0,1,…} are the set of non-negative integers and integers, respectively. It can be supposed that $\left\{\varepsilon_{t},t\in Z\right\}$ is a sequence of i.i.d. random variables with a mean of one and finite variance of $\sigma^{2}$. The modified multiplicative thinning-based INARCH (denoted by the MthINARCH(q)) model, which we deal with in this paper, is defined by

(2) $$X_{t}=\lambda_{t}\varepsilon_{t},\;\;\;\lambda_{t}=\omega \circ m+\sum_{i=1}^{q}\alpha_{i}\circ X_{t-i},$$

where 0<ω≤1, $0\leq \alpha_{i}<1,i=1,\ldots ,q$, *m* is a fixed positive integer number. In real applications, we can set *m* as the upper integer part of the sample mean. It is assumed that the Bernoulli terms corresponding to the binomial variables ω∘m and $\alpha_{i}\circ X_{t-i}$ are mutually independent and independent of the sequence $\left\{\varepsilon_{t},t\in Z\right\}$. The reason that we defined the new model in this way can be explained as follows. The additive term 1 in $\lambda_{t}$ and in (1) is unnatural, and is posed to ensure $\lambda_{t}>0$, but we can achieve this by adjusting the range of ω; therefore, we adopted a simple version of $\lambda_{t}$ in (2).

Now that we discuss the conditional mean and conditional variance of $X_{t}$. Note that $F_{t-1}$ is the σ-field generated by $X_{t-1},X_{t-2},\ldots$. For $E(\varepsilon_{t})=1$, let $\mu_{t}:=E\left(X_{t}|F_{t-1}\right)=E\left(\lambda_{t}\varepsilon_{t}|F_{t-1}\right)=E\left(\varepsilon_{t}\right)E\left(\lambda_{t}|F_{t-1}\right)=E\left(\lambda_{t}|F_{t-1}\right)=\omega m+\sum_{i=1}^{q}\alpha_{i}X_{t-i}$. Then we can obtain the conditional variance; first, let $\nu_{t}:=\mathrm{Var}\left(\lambda_{t}|F_{t-1}\right)$ and $\sigma_{t}^{2}:=\mathrm{Var}\left(X_{t}|F_{t-1}\right)$. For $E\left(\varepsilon_{t}\right)=1,\mathrm{Var}\left(\varepsilon_{t}\right)=\sigma^{2}$, so $E\left(\varepsilon_{t}^{2}\right)=\sigma^{2}+1$. Therefore,

$$\begin{array}{cc} \nu_{t}: & =\mathrm{Var}\left(\lambda_{t}|F_{t-1}\right)=\omega \left(1-\omega \right)m+\sum_{i=1}^{q}\alpha_{i}\left(1-\alpha_{i}\right)X_{t-i},\\ \sigma_{t}^{2}: & =\mathrm{Var}\left(X_{t}|F_{t-1}\right)=E\left(X_{t}^{2}|F_{t-1}\right)-{\left[E\left(X_{t}|F_{t-1}\right)\right]}^{2}=E\left(\lambda_{t}^{2}|F_{t-1}\right)E\left(\varepsilon_{t}^{2}\right)-\mu_{t}^{2}\\ \; & =\left[\mathrm{Var}\left(\lambda_{t}|F_{t-1}\right)+{\left(E\left(\lambda_{t}|F_{t-1}\right)\right)}^{2}\right]E\left(\varepsilon_{t}^{2}\right)-\mu_{t}^{2}\\ \; & =\left(\sigma^{2}+1\right)\left(\nu_{t}+\mu_{t}^{2}\right)-\mu_{t}^{2}=\left(\sigma^{2}+1\right)\nu_{t}+\sigma^{2}\mu_{t}^{2}. \end{array}$$

**Proposition** **1.** *The necessary and sufficient condition for the first-order stationarity of $X_{t}$ defined in (2) is that all roots of $1-\sum_{i=1}^{q}\alpha_{i}z^{i}=0$ should lie outside the unit circle.*

**Proposition** **2.** *The necessary and sufficient condition for the second-order stationarity of $X_{t}$ defined in (2) is that $\left(\sigma^{2}+1\right)\sum_{i=1}^{q}\alpha_{i}^{2}<1.$*

Proofs of Propositions 1 and 2 are similar to the proofs of Theorems 2.1 and 2.2 in Aknouche and Scotto (2022) [14], so we omit the details.

For convenience, we need to specify the distribution of $\left\{\varepsilon_{t}\right\}$ in (2). First, we let $\varepsilon_{t}∼P\left(1\right)$, then $E\left(\varepsilon_{t}\right)=\mathrm{Var}\left(\varepsilon_{t}\right)=1$, and this model is denoted by PMthINARCH(q). It is easy to obtain

$$\mu_{t}=\omega m+\sum_{i=1}^{q}\alpha_{i}X_{t-i},\;\;\;\sigma_{t}^{2}=2\nu_{t}+\mu_{t}^{2}.$$

Second, let $\varepsilon_{t}∼Ge\left(p^{*}\right)$. The mean of $\varepsilon_{t}$ is $\left(1-p^{*}\right)/p^{*}=1$, so we have $p^{*}=0.5$ and the variance is $\mathrm{Var}(\varepsilon_{t})=2$. This model is denoted by GMthINARCH(q), then we have

$$\mu_{t}=\omega m+\sum_{i=1}^{q}\alpha_{i}X_{t-i},\;\;\;\sigma_{t}^{2}=3\nu_{t}+2\mu_{t}^{2}.$$

## 3. Parameter Estimation

In this section, we will consider the SPMLE and its asymptotic properties, and a simulation study will be conducted to assess the performance of this estimator.

### 3.1. Saddlepoint Maximum Likelihood Estimation

Let $\theta ={\left(\omega ,\alpha_{1},\ldots ,\alpha_{q}\right)}^{T}$ be the unknown parameter vector. Note that according to the condition on $\varepsilon_{t}$, $\sigma^{2}$ is no longer an unknown parameter. The maximum likelihood estimator of θ was obtained by maximizing the conditional log-likelihood function

(3) $$l\left(\theta \right)=\sum_{t=1}^{n}\log P\left(X_{t}=x_{t}|X_{t-1}=x_{t-1},\ldots ,X_{t-q}=x_{t-q}\right),$$

giving $\hat{\theta }=\arg\max_{\theta }l\left(\theta \right).$ But the above procedure is challenging to implement because it is difficult to give the likelihood function due to the thinning operations.

Now we discuss the SPMLE. The conditional moment generating function of $X_{t}$ is

$$\begin{array}{cc} E(e^{uX_{t}}|X_{t-1}=x_{t-1},\ldots ,X_{t-q}=x_{t-q}) & =E(e^{u\lambda_{t}\varepsilon_{t}}|X_{t-1}=x_{t-1},\ldots ,X_{t-q}=x_{t-q})\\ \; & =E(e^{u\left(\omega \circ m+\sum_{i=1}^{q}\alpha_{i}\circ X_{t-i}\right)\varepsilon_{t}}|X_{t-1}=x_{t-1},\ldots ,X_{t-q}=x_{t-q})\\ \; & =E\left(e^{u\left(\omega \circ m\right)\varepsilon_{t}}\right)\prod_{i=1}^{q}E\left(e^{u\left(\alpha_{i}\circ x_{t-i}\right)\varepsilon_{t}}\right). \end{array}$$

**Remark** **1.** *Here we just consider the INARCH model instead of the INGARCH model because for the INGARCH model, the conditional cumulant-generating function of $X_{t}$ should be given by $E\left(e^{uX_{t}}|X_{t-1}=x_{t-1},\ldots ,X_{t-q}=x_{t-q}\right)=E\left(e^{u\left(\omega \circ m+\sum_{i=1}^{q}\alpha_{i}\circ X_{t-i}+\sum_{j=1}^{p}\beta_{j}\circ \lambda_{t-i}\right)\varepsilon_{t}}|X_{t-1}=x_{t-1},\ldots ,X_{t-q}=x_{t-q}\right).$ Notice that $X_{t}$ and $\lambda_{t}$ are correlated, it is difficult and complex to show the conditional cumulant-generating function.*

Using the binomial theorem ${\left(a+b\right)}^{n}=\sum_{k=0}^{n}C_{n}^{k}a^{n-k}b^{k}$, we have

$$\begin{array}{cc} E(e^{u\left(\omega \circ m\right)\varepsilon_{t}}) & =E\left[E(e^{u\left(\omega \circ m\right)\varepsilon_{t}}|\varepsilon_{t})\right]=E{\left(\omega e^{u\varepsilon_{t}}+\left(1-\omega \right)\right)}^{m}\\ \; & =E\left[\sum_{r=0}^{m}C_{m}^{r}{\left(1-\omega \right)}^{r}\omega^{m-r}e^{u\left(m-r\right)\varepsilon_{t}}\right]=\sum_{r=0}^{m}C_{m}^{r}{\left(1-\omega \right)}^{r}\omega^{m-r}E\left(e^{u\left(m-r\right)\varepsilon_{t}}\right). \end{array}$$

Similarly, we also have

$$E\left(e^{u\left(\alpha_{i}\circ x_{t-i}\right)\varepsilon_{t}}\right)=\sum_{r=0}^{x_{t-i}}C_{x_{t-i}}^{r}{\left(1-\alpha_{i}\right)}^{r}\alpha_{i}^{x_{t-i}-r}E\left(e^{u\left(x_{t-i}-r\right)\varepsilon_{t}}\right).$$

Therefore, for the PMthINARCH(q) model, we have

$$\begin{array}{cc} E(e^{u\left(\omega \circ m\right)\varepsilon_{t}}) & =\sum_{r=0}^{m}C_{m}^{r}{\left(1-\omega \right)}^{r}\omega^{m-r}e^{\left(e^{u(m-r)}-1\right)},\\ E(e^{u\left(\alpha_{i}\circ x_{t-i}\right)\varepsilon_{t}}) & =\sum_{r=0}^{x_{t-i}}C_{x_{t-i}}^{r}{\left(1-\alpha_{i}\right)}^{r}\alpha_{i}^{x_{t-i}-r}e^{\left(e^{u(x_{t-i}-r)}-1\right)}, \end{array}$$

while for the GMthINARCH(q) model, we have

$$\begin{array}{cc} E(e^{u\left(\omega \circ m\right)\varepsilon_{t}}) & =\sum_{r=0}^{m}C_{m}^{r}{\left(1-\omega \right)}^{r}\omega^{m-r}\frac{1}{2-e^{u(m-r)}},\\ E(e^{u\left(\alpha_{i}\circ x_{t-i}\right)\varepsilon_{t}}) & =\sum_{r=0}^{x_{t-i}}C_{x_{t-i}}^{r}{\left(1-\alpha_{i}\right)}^{r}\alpha_{i}^{x_{t-i}-r}\frac{1}{2-e^{u(x_{t-i}-r)}}. \end{array}$$

Thus the conditional cumulant-generating function of $X_{t}$ is:

$$\begin{array}{c} K_{t}\left(u\right)=\log\left[E\left(e^{uX_{t}}|X_{t-1}=x_{t-1},\ldots ,X_{t-q}=x_{t-q}\right)\right]=\log E\left(e^{u\left(\omega \circ m\right)\varepsilon_{t}}\right)+\sum_{i=1}^{q}\log E\left(e^{u\left(\alpha_{i}\circ x_{t-i}\right)\varepsilon_{t}}\right). \end{array}$$

A highly accurate approximation to the conditional mass function of $X_{t}$ at $x_{t}$ is provided by the saddlepoint approximation:

(4) $${\tilde{f}}_{X_{t}|X_{t-1}=x_{t-1},\ldots ,X_{t-q}=x_{t-q}}\left(x_{t}\right)={\left(2\pi K_{t}^{\prime \prime }\left({\tilde{u}}_{t}\right)\right)}^{-\frac{1}{2}}\exp\left\{K_{t}\left({\tilde{u}}_{t}\right)-{\tilde{u}}_{t}x_{t}\right\},$$

where ${\tilde{u}}_{t}$ is the unique value of *u* which satisfies the saddlepoint equation $K_{t}^{\prime }\left(u\right)=x_{t},$ with $K_{t}^{\prime }$ and $K_{t}^{\prime \prime }$ represent the first and second order derivatives of $K_{t}$ with respect to *u*. Notice that it is difficult to solve the saddlepoint equation $K_{t}^{\prime }\left(u\right)=x_{t}$ analytically; similar to that mentioned in Pedeli et al. (2015) [19], we can use the Newton–Raphson method to solve this equation.

The log-likelihood function (3) can be approximated by summing the logarithms of the corresponding density approximations (4), yielding:

(5) $${\tilde{L}}_{n}\left(\theta \right)=\sum_{t=1}^{n}{\tilde{l}}_{t}\left(\theta \right):=\sum_{t=1}^{n}\log{\tilde{f}}_{X_{t}|X_{t-1}=x_{t-1},\ldots ,X_{t-q}=x_{t-q}}\left(x_{t}\right).$$

The value θ maximizing this expression is called the saddlepoint maximum likelihood estimator (SPMLE).

### 3.2. Asymptotic Properties of the SPMLE

Now we discuss the asymptotic properties of the SPMLE. First we give the first-order Taylor expansion of $K_{t}^{\prime }\left(u\right)$ at u=0 yields,

(6) $$K_{t}^{\prime }\left(u\right)=K_{t}^{\prime }\left(0\right)+uK_{t}^{\prime \prime }\left(0\right)+o\left(u\right)=\mu_{t}\left(\theta \right)+u\sigma_{t}^{2}\left(\theta \right)+o\left(u\right),$$

where $\mu_{t}\left(\theta \right)$ and $\sigma_{t}^{2}\left(\theta \right)$ are the conditional mean and conditional variance of $X_{t}$. Notice that ${\tilde{u}}_{t}$ can be given by $K_{t}^{\prime }\left({\tilde{u}}_{t}\right)=x_{t}$, so with the Taylor series expansion of $K_{t}^{\prime }\left(u\right)$ in (6), we have:

(7) $${\tilde{u}}_{t}=\frac{x_{t}-\mu_{t}\left(\theta \right)}{\sigma_{t}^{2}\left(\theta \right)}+o\left(1\right),\;\;\;t=q+1,\ldots ,n.$$

Then, we can obtain the second-order Taylor expansion of $K_{t}\left(u\right)$ at u=0, which is:

(8) $$K_{t}\left(u\right)\approx uK_{t}^{\prime }\left(0\right)+\frac{u^{2}}{2}K_{t}^{\prime \prime }\left(0\right)=u\mu_{t}\left(\theta \right)+\frac{u^{2}}{2}\sigma_{t}^{2}\left(\theta \right).$$

Focusing on the exponent of the saddlepoint approximation (4), Equation (8) gives

$$K_{t}\left(u\right)-ux_{t}\approx u\left(\mu_{t}\left(\theta \right)-x_{t}\right)+\frac{u^{2}}{2}\sigma_{t}^{2}\left(\theta \right).$$

Then using Equation (7), we have

(9) $$K_{t}\left({\tilde{u}}_{t}\right)-{\tilde{u}}_{t}x_{t}\approx -\frac{{\left[x_{t}-\mu_{t}\left(\theta \right)\right]}^{2}}{2\sigma_{t}^{2}\left(\theta \right)}.$$

Hence, we can derive from (8) and (9) that the first-order saddlepoint approximation to the conditional probability mass function is approximately:

$$\begin{array}{cc} \; & {\tilde{f}}_{X_{t}|X_{t-1}=x_{t-1},\ldots ,X_{t-q}=x_{t-q}}\left(x_{t}\right)={\left(2\pi K_{t}^{\prime \prime }\left({\tilde{u}}_{t}\right)\right)}^{-\frac{1}{2}}\\ \; & \;\;\;\times \exp\left[-\frac{{\left(x_{t}-\omega m-\sum_{i=1}^{q}\alpha_{i}x_{t-i}\right)}^{2}}{2\left[\left(\sigma^{2}+1\right)\left(\omega \left(1-\omega \right)m+\sum_{i=1}^{q}\alpha_{i}\left(1-\alpha_{i}\right)x_{t-i}\right)+\sigma^{2}{\left(\omega m+\sum_{i=1}^{q}\alpha_{i}x_{t-i}\right)}^{2}\right]}\right]. \end{array}$$

Therefore, ${\tilde{L}}_{n}\left(\theta \right)=\sum_{t=1}^{n}{\tilde{l}}_{t}\left(\theta \right)=\sum_{t=1}^{n}\log{\tilde{f}}_{X_{t}|X_{t-1}=x_{t-1},\ldots ,X_{t-q}=x_{t-q}}\left(x_{t}\right)$ is the quasi-likelihood function for the estimation of θ. To establish the large-sample properties, we have

$$L_{n}\left(\theta \right)=\sum_{t=1}^{n}l_{t}\left(\theta \right)=\sum_{t=1}^{n}\log f_{X_{t}|X_{t-1}=x_{t-1},\ldots ,X_{t-q}=x_{t-q}}\left(x_{t}\right),$$

which is the ergodic approximation of ${\tilde{L}}_{n}\left(\theta \right)$. The first and second derivatives of the quasi-likelihood function are given in the Appendix A. The strong convergence and asymptotic normality for the SPMLE ${\hat{\theta }}_{n}$ are established in the following theorems.

First of all, the assumptions for Theorems 1 and 2 are listed as follows.

**Assumption** **1.** *The solution of the MthINARCH process is strictly stationary and ergodic.*

**Assumption** **2.** Θ *is compact and $\theta_{0}\in \overset{{}^{\circ}}{\Theta }$, where $\overset{{}^{\circ}}{\Theta }$ denotes the interior of* Θ. *For technical reasons, we assumed the lower and upper values of each component of parameters as $0<\omega_{L}\leq \omega \leq \omega_{U}\leq 1$ and $0\leq \alpha_{L}\leq \alpha_{i}\leq \alpha_{U}<1$, i=1,…,q.*

**Theorem** **1.** *Let ${\hat{\theta }}_{n}$ be a sequence of SPMLEs satisfying ${\hat{\theta }}_{n}=\arg\max_{\theta \in \Theta }{\tilde{L}}_{n}\left(\theta \right)$, then under Assumptions 1 and 2, ${\hat{\theta }}_{n}$ converges to $\theta_{0}$ almost as surely, as n→∞.*

**Theorem** **2.** *Under Assumptions 1 and 2, there exists a sequence of maximizers ${\hat{\theta }}_{n}$ of ${\tilde{L}}_{n}\left(\theta \right)$ such as that of n→∞,*

$$\sqrt{n}\left({\hat{\theta }}_{n}-\theta_{0}\right)\overset{d}{⟶}N\left(0,\Sigma^{-1}\right),$$

*where*

$$\Sigma =-E_{\theta_{0}}\left(\frac{\partial^{2}l_{t}\left(\theta_{0}\right)}{\partial \theta \partial \theta^{T}}\right),$$

*and* Σ *is positively definite.*

### 3.3. Simulation Study

In this section, simulation studies of PMthINARCH(q) and GMthINARCH(q) models for finite sample size are given, where q=2. Here, we used several combinations to show the performance of SPMLE, and the mean absolute deviation error (MADE) $\frac{1}{s}\sum_{j=1}^{s}\left|\hat{\theta_{j}}-\theta_{j}\right|$ was used as the evaluation criterion; here, *s* is the number of replications. The sample size is n=100,200,500, and the number of replications is s=200. We used the following combinations of ${\left(\omega ,\alpha_{1},\alpha_{2}\right)}^{T}$ as the true values to generate the random sample: A1 $={\left(0.65,0.4,0.4\right)}^{T}$, A2 $={\left(0.9,0.5,0.3\right)}^{T}$ for the PMthINARCH(2) model, and B1 $={\left(0.8,0.4,0.4\right)}^{T}$, B2 $={\left(0.65,0.3,0.5\right)}^{T}$ for the GMthINARCH(2) model. Table 1 and Table 2 show the results of these simulations. Notice that as the sample sizes become larger, the MADEs become smaller, and the estimates seem to be close to the true values. Therefore, the SPMLE performs well.

## 4. A Real Example

**Figure 1:**
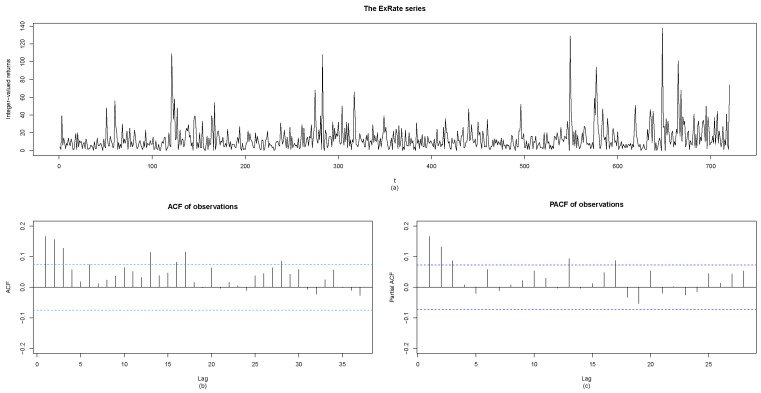
(**a**) The plot of integer-valued series of ExRate. (**b**) The plot of ACF of observations. (**c**) The plot of PACF of observations.

Here, we considered the number of tick changes by the minute of the euro to the British pound exchange rate (ExRate for short) on December 12th from 9.00 a.m. to 9.00 p.m. The dataset is available at the website http://www.histdata.com/ (accessed on 17 January 2023). The series comprises of 720 observations with a sample mean of 13.2153 and a sample variance of 224.2498. Obviously, the sample variance is much larger than the sample mean, which shows high overdispersion, and this high overdispersion can also be seen in Figure 1a. Figure 1b,c are the plots of the autocorrelation function (ACF), and the partial autocorrelation function (PACF) means that we know the tick changes are correlated.

We analyzed the data using the PMthINARCH(3) model, GMthINARCH(3) model, Poisson INAR(3) (here denoted by PINAR(3) for short) model, and the INARCH(3) model. The Poisson INAR model is mentioned in Pedeli et al. (2015) [19], and the SPMLE was used to estimate the parameters. Here, the innovations in the PINAR model were assumed to be Poisson with a mean of one. The INARCH model with a Poisson deviate was proposed by Ferland et al. (2006) [5], and the MLE was used to estimate the parameters. According to Aknouche and Scotto (2022) [14], in real applications, we can set *m* as the upper integer part of the sample mean. Here the sample mean is 13.2153, so *m* is set to the value of 14. Table 3 gives the estimates of SPMLE and the values of the Akaike information criterion (AIC) and Bayesian information criterion (BIC). According to Table 3, it is clear to see that the values of AIC and BIC of PMthINARCH(3) and GMthINARCH(3) are smaller than those of the PINAR(3) and INARCH(3) models, the values of AIC and BIC of INARCH(3) are smaller than those of the PINAR(3) model. Moreover, the values of AIC and BIC of PMthINARCH(3) are smaller than those of GMthINARCH(3). In summary, the INARCH model performed better than the PINAR model; meanwhile, the PMthINARCH model and GMthINARCH model performed better than the PINAR model and INARCH model.

According to Aknouche and Scotto (2022) [14], the two-stage weighted least squares estimation (2SWLSE) was used to estimate the parameters of the MthINGARCH model. Therefore, to compare the performance of 2SWLSE and SPMLE, and the performance of PMthINARCH, GMthINARCH, and PINAR models, to consider the in-sample and out-of-sample forecasts of these two estimation methods and the three models above, respectively. First, we considered the in-sample forecast. We used all of the observations to estimate the model, and then we could forecast the last 10 observations 711–720, the last 15 observations 706–720, and the last 20 observations 701–720; these three-time horizons of in-sample forecast are denoted by C1, C2, and C3, respectively. Similar to the in-sample forecast process, we also considered the out-of-sample forecast and divided all the observations into three-time horizons: the first one was 1–710 and 711–720, the second one was 1–705 and 706–720, and the third one was 1–700 and 701–720, which are denoted by D1, D2, and D3, respectively.

Here we illustrate the performance of the considered models by comparing the MADEs of each forecast. The MADEs of in-sample forecasts and out-of-sample forecasts for three models with SPMLE are shown in Table 4. The MADEs of the in-sample forecasts and out-of-sample forecasts for the PMthINARCH model with 2SWLSE and SPMLE are shown in Table 5, and the in-sample forecasts and out-of-sample forecasts for the GMthINARCH model with 2SWLSE and SPMLE are shown in Table 6. According to Table 4, the MADEs of PMthINARCH(3) and GMthINARCH(3) are smaller than those of PINAR(3), Table 5 and Table 6 show that the MADEs of PMthINARCH(3) and GMthINARCH(3) of SPMLE are smaller than those of 2SWLSE; meanwhile, in these three Tables, the MADEs of in-sample forecasts were smaller than those of out-of-sample forecasts. In summary, the PMthINARCH model and GMthINARCH model were superior to the PINAR model in modeling this real data set, and the PMthINARCH model performed better than the GMthINARCH model. Meanwhile, the performance of SPMLE was better than 2SWLSE for MthINARCH models.

## 5. Conclusions

In this paper, we modified a multiplicative thinning-based INARCH model. The probability mass function of random variables is provided by saddlepoint approximation. We used the SPMLE to estimate the parameters and obtain the asymptotic distribution of the SPMLE. Moreover, to show the superiority of the MthINARCH models and the SPMLE, we used the PMthINARCH(q) process and GMthINARCH(q) process for discussion and comparison. The SPMLE performs well in the simulation studies. A real dataset indicates that the PMthINARCH model and the GMthINARCH model are able to describe the overdispersed integer-valued data, and the real data example leads to a superior performance of the MthINARCH models compared with the PINAR and INARCH models. In addition, the results also show a superior performance of SPMLE compared with 2SWLSE.

For further discussion, more research is needed for some aspects. Here we used the Poisson distribution and geometric distribution for $\varepsilon_{t}$; however, we could use the negative binomial distribution or some zero-inflated distributions as well. Moreover, we just considered the INARCH model, so the corresponding INGARCH model should be considered as well.

## Figures and Tables

**Table 1:** Mean and MADE of estimates for PMthINARCH(2) model with SPMLE.

Model				ω	$\alpha_{1}$	$\alpha_{2}$
A1	*m* = 3	*n* = 100	Mean	0.6069	0.5356	0.3569
			MADE	0.3681	0.2866	0.2510
		*n* = 200	Mean	0.5722	0.5026	0.3952
			MADE	0.3557	0.2434	0.2243
		*n* = 500	Mean	0.6436	0.4888	0.4140
			MADE	0.2724	0.1287	0.1005
A2	*m* = 8	*n* = 100	Mean	0.7782	0.5076	0.4750
			MADE	0.2533	0.2752	0.3007
		*n* = 200	Mean	0.7935	0.5161	0.4701
			MADE	0.2318	0.2527	0.2778
		*n* = 500	Mean	0.8703	0.5170	0.4677
			MADE	0.1752	0.2155	0.2390

**Table 2:** Mean and MADE of estimates for GMthINARCH(2) model with SPMLE.

Model				ω	$\alpha_{1}$	$\alpha_{2}$
B1	*m* = 4	*n* = 100	Mean	0.7821	0.2930	0.2870
			MADE	0.1195	0.1499	0.1766
		*n* = 200	Mean	0.8190	0.3611	0.3185
			MADE	0.1121	0.1425	0.1640
		*n* = 500	Mean	0.8456	0.3610	0.3298
			MADE	0.0601	0.1331	0.1414
B2	*m* = 6	*n* = 100	Mean	0.4718	0.2086	0.3811
			MADE	0.1965	0.1466	0.1463
		*n* = 200	Mean	0.5186	0.2632	0.5080
			MADE	0.1607	0.1198	0.1412
		*n* = 500	Mean	0.5468	0.2874	0.4896
			MADE	0.1415	0.1050	0.0770

**Table 3:** Estimation results: AIC and BIC values for PMthINARCH(3), GMthINARCH(3), PINAR(3) and INARCH(3) models.

PMthINARCH(3)	ω	$\alpha_{1}$	$\alpha_{2}$	$\alpha_{3}$	AIC	BIC
	0.3242	0.5214	0.1945	0.0842	1395.296	1413.613
GMthINARCH(3)	ω	$\alpha_{1}$	$\alpha_{2}$	$\alpha_{3}$	AIC	BIC
	0.4904	0.2532	0.2155	0.2392	1402.472	1420.789
PINAR(3)	$\alpha_{1}$	$\alpha_{2}$	$\alpha_{3}$		AIC	BIC
	0.1335	0.4116	0.3901		1572.806	1586.544
INARCH(3)	ω	$\alpha_{1}$	$\alpha_{2}$	$\alpha_{3}$	AIC	BIC
	8.5670	0.1140	0.1379	0.1009	1524.638	1542.955

**Table 4:** MADEs of in-sample forecasts and out-of-sample forecasts for PMthINARCH(3), GMthINARCH(3), and PINAR(3) models with SPMLE.

Methods of Forecast		PMthINARCH	GMthINARCH	PINAR
In-sample	C1	15.30	16.80	17.40
	C2	15.87	17.67	18.40
	C3	16.65	20.70	21.90
Out-of-sample	D1	17.50	17.70	22.50
	D2	19.47	19.80	23.80
	D3	20.50	25.25	27.50

**Table 5:** MADEs of in-sample forecasts and out-of-sample forecasts for PMthINARCH(3) model with SPMLE and 2SWLSE.

Methods of Forecast		SPMLE	2SWLSE
In-sample	C1	15.30	16.20
	C2	15.87	17.20
	C3	16.65	18.55
Out-of-sample	D1	17.50	18.60
	D2	19.47	21.67
	D3	20.50	22.70

**Table 6:** MADEs of in-sample forecasts and out-of-sample forecasts for GMthINARCH(3) model with SPMLE and 2SWLSE.

Methods of Forecast		SPMLE	2SWLSE
In-sample	C1	16.80	17.20
	C2	17.67	18.07
	C3	20.70	21.05
Out-of-sample	D1	17.70	19.90
	D2	19.80	22.87
	D3	25.25	26.50

## Data Availability

The dataset is available at the website http://www.histdata.com/ (accessed on 17 January 2023).

## Appendix A

### Appendix A.1. Details of SPMLE

Here, we give the derivatives of $K_{t}\left(u\right)$ mentioned in Section 3.1 of PMthINARCH(q) and GMthINARCH(q). Now we give $K_{t}^{\prime }\left(u\right)$ and $K_{t}^{\prime \prime }\left(u\right)$ of PMthINARCH(q). In Section 3.1, we have

$$K_{t}\left(u\right)=\log E\left(e^{u\left(\omega \circ m\right)\varepsilon_{t}}\right)+\sum_{i=1}^{q}\log E\left(e^{u\left(\alpha_{i}\circ x_{t-i}\right)\varepsilon_{t}}\right)=\log a_{1}+\sum_{i=1}^{q}\log b_{1},$$

so the derivatives of $K_{t}\left(u\right)$ are given by

$$\begin{array}{c} K_{t}^{\prime }\left(u\right)=\frac{c_{1}}{a_{1}}+\sum_{i=1}^{q}\frac{d_{1}}{b_{1}},\;\;\;K_{t}^{\prime \prime }\left(u\right)=\frac{e_{1}a_{1}-c_{1}^{2}}{a_{1}^{2}}+\sum_{i=1}^{q}\frac{f_{1}b_{1}-d_{1}^{2}}{b_{1}^{2}}, \end{array}$$

where

$$\begin{array}{cc} \; & a_{1}=\sum_{r=0}^{m}C_{m}^{r}{\left(1-\omega \right)}^{r}\omega^{m-r}e^{e^{u(m-r)}-1},\\ \; & b_{1}=\sum_{r=0}^{x_{t-i}}C_{x_{t-i}}^{r}{\left(1-\alpha_{i}\right)}^{r}\alpha_{i}^{x_{t-i}-r}e^{e^{u(x_{t-i}-r)}-1},\\ \; & c_{1}=\sum_{r=0}^{m}C_{m}^{r}{\left(1-\omega \right)}^{r}\omega^{m-r}e^{u(m-r)}e^{e^{u(m-r)}-1},\\ \; & d_{1}=\sum_{r=0}^{x_{t-i}}C_{x_{t-i}}^{r}{\left(1-\alpha_{i}\right)}^{r}\alpha_{i}^{x_{t-i}-r}e^{u(x_{t-i}-r)}e^{e^{u(x_{t-i}-r)}-1},\\ \; & e_{1}=\sum_{r=0}^{m}C_{m}^{r}{\left(1-\omega \right)}^{r}\omega^{m-r}e^{u(m-r)}{\left(m-r\right)}^{2}e^{e^{u(m-r)}-1}\left[1+e^{u(m-r)}\right],\\ \; & f_{1}=\sum_{r=0}^{x_{t-i}}C_{x_{t-i}}^{r}{\left(1-\alpha_{i}\right)}^{r}\alpha_{i}^{x_{t-i}-r}{\left(x_{t-i}-r\right)}^{2}e^{u(x_{t-i}-r)}e^{e^{u(x_{t-i}-r)}-1}\left[1+e^{u(x_{t-i}-r)}\right]. \end{array}$$

Then we give $K_{t}^{\prime }\left(u\right)$ and $K_{t}^{\prime \prime }\left(u\right)$ of GMthINARCH(q). In Section 3.1, we have

$$K_{t}\left(u\right)=\log E\left(e^{u\left(\omega \circ m\right)\varepsilon_{t}}\right)+\sum_{i=1}^{q}\log E\left(e^{u\left(\alpha_{i}\circ x_{t-i}\right)\varepsilon_{t}}\right)=\log a_{2}+\sum_{i=1}^{q}\log b_{2},$$

so the derivatives of $K_{t}\left(u\right)$ are given by

$$\begin{array}{c} K_{t}^{\prime }\left(u\right)=\frac{c_{2}}{a_{2}}+\sum_{t=1}^{q}\frac{d_{2}}{b_{2}},\;\;\;K_{t}^{\prime \prime }\left(u\right)=\frac{e_{2}a_{2}-c_{2}^{2}}{a_{2}^{2}}+\sum_{t=1}^{q}\frac{f_{2}b_{2}-d_{2}^{2}}{b_{2}^{2}}, \end{array}$$

where

$$\begin{array}{cc} \; & a_{2}=\sum_{r=0}^{m}C_{m}^{r}{\left(1-\omega \right)}^{r}\omega^{m-r}\frac{1}{2-(2-e^{u(m-r)})},\\ \; & b_{2}=\sum_{r=0}^{x_{t-i}}C_{x_{t-i}}^{r}{\left(1-\alpha_{i}\right)}^{r}\alpha_{i}^{x_{t-i}-r}\frac{1}{2-(2-e^{u(x_{t-i}-r)})},\\ \; & c_{2}=\frac{1}{4}\sum_{r=0}^{m}C_{m}^{r}{\left(1-\omega \right)}^{r}\omega^{m-r}\left(m-r\right)\frac{e^{u(m-r)}}{{\left[1-\left(1-\frac{1}{2}e^{u(m-r)}\right)\right]}^{2}},\\ \; & d_{2}=\frac{1}{4}\sum_{r=0}^{x_{t-i}}C_{x_{t-i}}^{r}{\left(1-\alpha_{i}\right)}^{r}\alpha_{i}^{x_{t-i}-r}\left(x_{t-i}-r\right)\frac{e^{u(x_{t-i}-r)}}{{\left[1-\left(1-\frac{1}{2}e^{u(x_{t-i}-r)}\right)\right]}^{2}},\\ \; & e_{2}=\frac{1}{4}\sum_{r=0}^{m}C_{m}^{r}{\left(1-\omega \right)}^{r}\omega^{m-r}{\left(m-r\right)}^{2}e^{u(m-r)}\frac{1+\frac{1}{2}e^{u(m-r)}}{{\left[1-\left(1-\frac{1}{2}e^{u(m-r)}\right)\right]}^{3}},\\ \; & f_{2}=\frac{1}{4}\sum_{r=0}^{x_{t-i}}C_{x_{t-i}}^{r}{\left(1-\alpha_{i}\right)}^{r}\alpha_{i}^{x_{t-i}-r}{\left(x_{t-i}-r\right)}^{2}e^{u(x_{t-i}-r)}\frac{1+\frac{1}{2}e^{u(x_{t-i}-r)}}{{\left[1-\left(1-\frac{1}{2}e^{u(x_{t-i}-r)}\right)\right]}^{3}}. \end{array}$$

### Appendix A.2. Derivatives of the Quasi-Likelihood Function

The conditional log-quasi-likelihood function $l_{t}\left(\theta \right)$ is continuous on Θ: for 1≤t≤n,

$$\begin{array}{cc} \frac{\partial l_{t}\left(\theta \right)}{\partial \theta } & =m_{1}\frac{\partial \mu_{t}\left(\theta \right)}{\partial \theta }+m_{2}\frac{\partial \sigma_{t}^{2}\left(\theta \right)}{\partial \theta },\\ \frac{\partial^{2}l_{t}\left(\theta \right)}{\partial \theta \partial \theta^{T}} & =\left(m_{1}-m_{3}\right)\frac{\partial^{2}\mu_{t}\left(\theta \right)}{\partial \theta \partial \theta^{T}}-2m_{1}m_{3}\frac{\partial \mu_{t}\left(\theta \right)}{\partial \theta }\frac{\partial \sigma_{t}^{2}\left(\theta \right)}{\partial \theta^{T}}+\left(m_{2}+\frac{m_{3}^{2}}{2}-m_{1}^{2}m_{3}\right)\frac{\partial^{2}\sigma_{t}^{2}\left(\theta \right)}{\partial \theta \partial \theta^{T}}, \end{array}$$

where

$$m_{1}=\frac{X_{t}-\mu_{t}\left(\theta \right)}{\sigma_{t}^{2}\left(\theta \right)},\;\;\;m_{2}=\frac{{\left(X_{t}-\mu_{t}\left(\theta \right)\right)}^{2}-\sigma_{t}^{2}\left(\theta \right)}{2\sigma_{t}^{4}\left(\theta \right)},\;\;\;m_{3}=\frac{1}{\sigma_{t}^{2}\left(\theta \right)}.$$

Then the first and second derivatives of $\mu_{t}\left(\theta \right)$ and $\sigma_{t}^{2}\left(\theta \right)$ can be easily expressed by

$$\begin{array}{cc} \frac{\partial \mu_{t}\left(\theta \right)}{\partial \omega } & =m,\;\;\;\frac{\partial \mu_{t}\left(\theta \right)}{\partial \alpha_{i}}=X_{t-i},\\ \frac{\partial \sigma_{t}^{2}\left(\theta \right)}{\partial \omega } & =\left(\sigma^{2}+1\right)\left(m-2\omega m\right)+2\sigma^{2}\left(m^{2}\omega +m\sum_{i=1}^{q}\alpha_{i}X_{t-i}\right),\\ \frac{\partial \sigma_{t}^{2}\left(\theta \right)}{\partial \alpha_{i}} & =\left(\sigma^{2}+1\right)\left(X_{t-i}-2\alpha_{i}X_{t-i}\right)+2\sigma^{2}\left(m\omega X_{t-i}+\alpha_{i}X_{t-i}^{2}\right),\\ \frac{\partial^{2}\mu_{t}\left(\theta \right)}{\partial \omega^{2}} & =0,\;\;\;\frac{\partial^{2}\mu_{t}\left(\theta \right)}{\partial \alpha_{i}^{2}}=1,\;\;\;\frac{\partial^{2}\mu_{t}\left(\theta \right)}{\partial \omega \alpha_{i}}=0,\\ \frac{\partial^{2}\sigma_{t}^{2}\left(\theta \right)}{\partial \omega^{2}} & =-2m\left(\sigma^{2}+1\right)+2m^{2}\sigma^{2},\;\;\;\frac{\partial^{2}\sigma_{t}^{2}\left(\theta \right)}{\partial \alpha_{i}^{2}}=-2X_{t-i}\left(\sigma^{2}+1\right)+2X_{t-i}^{2}\sigma^{2},\\ \frac{\partial^{2}\sigma_{t}^{2}\left(\theta \right)}{\partial \omega \alpha_{i}} & =2m\sigma^{2}X_{t-i}. \end{array}$$

### Appendix A.3. Proof of Theorem 1

The techniques used here are mainly based on Francq and Zakoïan (2004) [20]. We will establish the following intermediate results:

(i) $\lim_{n\to \infty }\sup_{\theta \in \Theta }\left|\frac{1}{n}\left(L_{n}\left(\theta \right)-{\tilde{L}}_{n}\left(\theta \right)\right)\right|=0\;\;\;a.s.$
(ii) $E(l_{t}\left(\theta \right))$ is continuous in θ.
(iii) It exists t∈Z such that $\sigma_{t}^{2}\left(\theta \right)=\sigma_{t}^{2}\left(\theta_{0}\right)$ a.s., then $\Rightarrow \theta =\theta_{0}$.
(iv) Any $\theta \neq \theta_{0}$ has a neighbourhood V(θ) such that
  $$\underset{n\to \infty }{lim sup}\underset{\theta^{*}\in V_{k}\left(\theta \right)\cap \Theta }{\sup}\frac{1}{n}{\tilde{L}}_{n}\left(\theta^{*}\right)>E_{\theta_{0}}l_{1}\left(\theta_{0}\right)\;\;\;a.s.$$

First we prove (i). Let $at:=\sup_{\theta \in \Theta }\left|{\tilde{\mu }}_{t}\left(\theta \right)-\mu_{t}\left(\theta \right)\right|$, $b_{t}:=\sup_{\theta \in \Theta }\left|{\tilde{\sigma }}_{t}^{2}\left(\theta \right)-\sigma_{t}^{2}\left(\theta \right)\right|.$ Standard arguments from Corollary 2.2 in Aknouche and Francq (2023) [21] show that $a_{t}\left(1+X_{t}+\sup_{\theta \in \Theta }\mu_{t}\left(\theta \right)\right)\to 0,a.s.$ and $b_{t}\left(1+X_{t}^{2}+\sup_{\theta \in \Theta }\mu_{t}^{2}\left(\theta \right)\right)\to 0,a.s.,t\to \infty ,$ so we obtain the inequality

$$\begin{array}{cc} \; & \underset{\theta \in \Theta }{\sup}\left|\frac{1}{n}\left(L_{n}\left(\theta \right)-{\tilde{L}}_{n}\left(\theta \right)\right)\right|=\underset{\theta \in \Theta }{\sup}\left|\frac{1}{2n}\sum_{t=1}^{n}\log\frac{{\tilde{\sigma }}_{t}^{2}\left(\theta \right)}{\sigma_{t}^{2}\left(\theta \right)}+\left(\frac{{\left(x_{t}-{\tilde{\mu }}_{t}\right)}^{2}}{{\tilde{\sigma }}_{t}^{2}}-\frac{{\left(x_{t}-\mu_{t}\left(\theta \right)\right)}^{2}}{\sigma_{t}^{2}}\right)\right|\\ \; & \leq \underset{\theta \in \Theta }{\sup}\left|\frac{1}{2n}\sum_{t=1}^{n}\frac{{\tilde{\sigma }}_{t}^{2}\left(\theta \right)-\sigma_{t}^{2}\left(\theta \right)}{\sigma_{t}^{2}\left(\theta \right)}+\left(\frac{{\left(x_{t}-{\tilde{\mu }}_{t}\left(\theta \right)\right)}^{2}}{{\tilde{\sigma }}_{t}^{2}\left(\theta \right)}-\frac{{\left(x_{t}-\mu_{t}\left(\theta \right)\right)}^{2}}{\sigma_{t}^{2}}\right)\right|\\ \; & \leq \underset{\theta \in \Theta }{\sup}\frac{1}{2n}\sum_{t=1}^{n}\frac{|{\tilde{\sigma }}_{t}^{2}\left(\theta \right)-\sigma_{t}^{2}\left(\theta \right)|}{\sigma_{t}^{2}\left(\theta \right)}+\frac{|{\tilde{\mu }}_{t}\left(\theta \right)-\mu_{t}\left(\theta \right)||\mu_{t}\left(\theta \right)+{\tilde{\mu }}_{t}\left(\theta \right)-2X_{t}|}{{\tilde{\sigma }}_{t}^{2}\left(\theta \right)}\\ \; & +\frac{\left|{\tilde{\sigma }}_{t}^{2}\left(\theta \right)-\sigma_{t}^{2}\left(\theta \right)\right|{\left|X_{t}-\mu_{t}\left(\theta \right)\right|}^{2}}{\sigma_{t}^{2}\left(\theta \right){\tilde{\sigma }}_{t}^{2}\left(\theta \right)}\\ \; & \leq \frac{1}{2n}\sum_{t=1}^{n}\frac{2}{\sigma_{t}^{2}\left(\theta \right)}a_{t}\left(1+X_{t}+\underset{\theta \in \Theta }{\sup}\mu_{t}\left(\theta \right)\right)+\frac{1+{\tilde{\sigma }}_{t}^{2}\left(\theta \right)}{\sigma_{t}^{2}\left(\theta \right){\tilde{\sigma }}_{t}^{2}\left(\theta \right)}c_{t}\left(1+X_{t}^{2}+\underset{\theta \in \Theta }{\sup}\mu_{t}^{2}\left(\theta \right)\right). \end{array}$$

The a.s. limit holds because of the Cesàro lemma.

We prove (ii) now. For any θ∈Θ, let $V_{\eta }\left(\theta \right)=B\left(\theta ,\eta \right)$ be an open ball centered at θ with radius η,

$$\begin{array}{cc} \left|l_{t}\left(\tilde{\theta }\right)-l_{t}\left(\theta \right)\right| & \leq |\sigma_{t}^{2}\left(\tilde{\theta }\right)-\sigma_{t}^{2}\left(\theta \right)|\left|\frac{X_{t}^{2}+\mu_{t}^{2}\left(\theta \right)+\sigma_{t}^{2}\left(\tilde{\theta }\right)}{\sigma_{t}^{2}\left(\theta \right)\sigma_{t}^{2}\left(\tilde{\theta }\right)}\right|+\frac{|\mu_{t}\left(\tilde{\theta }\right)-\mu_{t}\left(\theta \right)\left|\right|\mu_{t}\left(\theta \right)+\mu_{t}\left(\tilde{\theta }\right)-2X_{t}|}{\sigma_{t}^{2}\left(\tilde{\theta }\right)}. \end{array}$$

Then

$$\begin{array}{cc} E\left(\underset{\tilde{\theta \in V_{\eta }\left(\theta \right)}}{\sup}\left|l_{t}\left(\tilde{\theta }\right)-l_{t}\left(\theta \right)\right|\right) & \leq \|\sigma_{t}^{2}\left(\tilde{\theta }\right)-\sigma_{t}^{2}\left(\theta \right){\|}_{2}{\left\|\frac{X_{t}^{2}+\mu_{t}^{2}\left(\theta \right)+\sigma_{t}^{2}\left(\tilde{\theta }\right)}{\sigma_{t}^{2}\left(\theta \right)\sigma_{t}^{2}\left(\tilde{\theta }\right)}\right\|}_{2}\\ \; & +\frac{\|\mu_{t}\left(\tilde{\theta }\right)-\mu_{t}\left(\theta \right){\|}_{2}{\left\|\mu_{t}\left(\theta \right)+\mu_{t}\left(\tilde{\theta }\right)-2X_{t}\right\|}_{2}}{\sigma_{t}^{2}\left(\tilde{\theta }\right)}\to 0,\;\;\;as\;\;\;\eta \to 0. \end{array}$$

Next, we check (iii). By Jensen’s inequality, we have

$$\begin{array}{cc} E\left[l_{t}\left(\theta \right)-l_{t}\left(\theta_{0}\right)\right] & =E\left[E\left(\frac{1}{2}\log\frac{\sigma_{t}^{2}\left(\theta_{0}\right)}{\sigma_{t}^{2}\left(\theta \right)}+\frac{{\left(x_{t}-\mu_{t}\left(\theta_{0}\right)\right)}^{2}}{2\sigma_{t}^{2}\left(\theta_{0}\right)}-\frac{{\left(x_{t}-\mu_{t}\left(\theta \right)\right)}^{2}}{2\sigma_{t}^{2}\left(\theta \right)}|F_{t-1}\right)\right]\\ \; & \leq E\left[\log E\left(\frac{\sigma_{t}^{2}\left(\theta_{0}\right)}{\sigma_{t}^{2}\left(\theta \right)}|F_{t-1}\right)\right]\\ \; & =E(\log(1))=0. \end{array}$$

The equality holds if $\frac{\sigma_{t}^{2}\left(\theta_{0}\right)}{\sigma_{t}^{2}\left(\theta \right)}=1$ a.s. $F_{t-1}$, i.e., $\theta =\theta_{0}$.

Then the proof of (iv) is similar to that in the Supplementary Material A.4 in Xu and Zhu (2022) [22]. Here we omit the details.

### Appendix A.4. Proof of the Positive Definiteness of *Σ*

Here, we prove the positive definiteness of Σ. By definition of positive definiteness, we need to prove for any $\xi ={\left(\xi_{0},\xi_{1},\ldots ,\xi_{q}\right)}^{T}\in R^{q+1},$ if $\xi^{T}\Sigma \xi =0,$ then ξ=0.

$$\begin{array}{cc} \xi^{T}\Sigma \xi & =\xi^{T}E\left[\frac{1}{2\sigma_{t}^{4}\left(\theta_{0}\right)}\frac{\partial \sigma_{t}^{2}\left(\theta_{0}\right)}{\partial \theta }\frac{\partial \sigma_{t}^{2}\left(\theta_{0}\right)}{\partial \theta^{T}}+\frac{1}{\sigma_{t}^{2}\left(\theta_{0}\right)}\frac{\partial \mu_{t}\left(\theta_{0}\right)}{\partial \theta }\frac{\partial \mu_{t}\left(\theta_{0}\right)}{\partial \theta^{T}}\right]\xi \\ \; & =E\left[\frac{1}{2\sigma_{t}^{4}\left(\theta_{0}\right)}{\left(\xi^{T}\frac{\partial \sigma_{t}^{2}\left(\theta_{0}\right)}{\partial \theta }\right)}^{2}+\frac{1}{\sigma_{t}^{2}\left(\theta_{0}\right)}{\left(\xi^{T}\frac{\partial \mu_{t}\left(\theta_{0}\right)}{\partial \theta }\right)}^{2}\right]. \end{array}$$

Suppose the left-hand side is 0, then under Assumption 1, the expectation in the right-hand side is 0 for any t∈Z. Because $\sigma_{t}^{2}\left(\theta_{0}\right)>0,$ this expectation is always greater than or equal to 0. It equals 0 only when $\xi^{T}\frac{\partial \sigma_{t}^{2}\left(\theta_{0}\right)}{\partial \theta }=0$ and $\xi^{T}\frac{\partial \mu_{t}\left(\theta_{0}\right)}{\partial \theta }=0$ almost surely. Thus, $\xi^{T}\Sigma \xi =0$ yields $\xi^{T}\frac{\partial \sigma_{t}^{2}\left(\theta_{0}\right)}{\partial \theta }=0$ and $\xi^{T}\frac{\partial \mu_{t}\left(\theta_{0}\right)}{\partial \theta }=0$ a.s. for t∈Z, and vice versa.

Using vector form of $\frac{\partial \sigma_{t}^{2}\left(\theta_{0}\right)}{\partial \theta }$, we have

$${\xi_{a}}^{T}\frac{\partial \sigma_{t}^{2}\left(\theta_{0}\right)}{\partial \theta }=\xi^{T}\left(\begin{array}{c} \left(\sigma^{2}+1\right)\left(m-2\omega m\right)+2\sigma^{2}\left(\omega m^{2}+m\sum_{i=1}^{q}\alpha_{i}X_{t-i}\right)\\ \left(\sigma^{2}+1\right)\left(X_{t-1}-2\alpha_{1}X_{t-1}\right)+2\sigma^{2}\left(\omega mX_{t-1}+\alpha_{1}X_{t-1}^{2}\right)\\ \vdots \\ \left(\sigma^{2}+1\right)\left(X_{t-q}-2\alpha_{q}X_{t-q}\right)+2\sigma^{2}\left(\omega mX_{t-q}+\alpha_{q}X_{t-q}^{2}\right) \end{array}\right).$$

Suppose the left-hand side is 0 almost surely, then the right-hand side is also 0 almost surely, which can be written as

$$\begin{array}{cc} \; & \xi_{0}\left(\sigma^{2}+1\right)\left(m-2\omega m\right)+2\sigma^{2}\xi_{0}\left(\omega m^{2}+m\sum_{i=1}^{q}\alpha_{i}X_{t-i}\right)\\ \; & +\xi_{1}\left(\sigma^{2}+1\right)\left(X_{t-1}-2\alpha_{1}X_{t-1}\right)+2\sigma^{2}\xi_{1}\left(\omega mX_{t-1}+\alpha_{1}X_{t-1}^{2}\right)+M_{t-2}=0\;\;a.s., \end{array}$$

where

$$M_{t-2}=\sum_{k=2}^{p}\xi_{k}\left[\left(\sigma^{2}+1\right)\left(X_{t-k}-2\alpha_{k}X_{t-k}\right)+2\sigma^{2}\left(\omega mX_{t-k}+\alpha_{k}X_{t-k}^{2}\right)\right].$$

So the coefficients of the above equation must satisfy

$$\begin{array}{cc} \; & \xi_{i}\left(\sigma^{2}+1\right)=0,\;\;\;2\sigma^{2}\xi_{i}=0,\;\;\;i=0,\ldots ,q. \end{array}$$

For $\sigma^{2}>0,$ we must have $\xi_{i}=0,i=0,\ldots ,q.$ Thus, $\xi ={\left(\xi_{0},\xi_{1},\ldots ,\xi_{q}\right)}^{T}=0,$ which completes the proof of the positive definiteness of Σ.

### Appendix A.5. Lemmas for the Proof of Theorem 2

Similar to the proof of Theorem 1.2 in Hu (2016) [9], we give some related lemmas for the proof of Theorem 2. According to the derivatives of the quasi-likelihood function, we have

$$\begin{array}{cc} \frac{\partial \mu_{t}\left(\theta \right)}{\partial \omega } & =m,\\ \frac{\partial \sigma_{t}^{2}\left(\theta \right)}{\partial \omega } & =\left(\sigma^{2}+1\right)\left(m-2\omega m\right)+2\sigma^{2}\left(m^{2}\omega +m\sum_{i=1}^{q}\alpha_{i}X_{t-i}\right),\\ \; & \leq \left(\sigma^{2}+1\right)m\left(1-2\omega_{L}\right)+2\sigma^{2}\left(m^{2}\omega_{U}+m\sum_{i=1}^{q}\alpha_{U}X_{t-i}\right), \end{array}$$

thus, $E{\left(\frac{\partial \mu_{t}\left(\theta \right)}{\partial \omega }\right)}^{2}<\infty$ and $E{\left(\frac{\partial \sigma_{t}^{2}\left(\theta \right)}{\partial \omega }\right)}^{2}<\infty$. Likewise for the other terms of parameters.

**Lemma** **A1.**
*Under Assumptions 1 and 2, when n→∞,*

$$\frac{1}{\sqrt{n}}\sum_{t=1}^{n}\frac{\partial {\tilde{l}}_{t}\left(\theta_{0}\right)}{\partial \theta_{i}}\overset{d}{⟶}N\left(0,\Sigma \right),\;\;\;\frac{1}{n}\sum_{t=1}^{n}\frac{\partial^{2}{\tilde{l}}_{t}\left(\theta_{0}\right)}{\partial \theta_{i}\partial \theta_{j}}\overset{P}{⟶}-\Sigma .$$

**Proof of Lemma** **A1.** First, we show that

$$n^{-1/2}\sum_{t=1}^{n}\left|\frac{\partial l_{t}\left(\theta_{0}\right)}{\partial \theta_{i}}-\frac{\partial {\tilde{l}}_{t}\left(\theta_{0}\right)}{\partial \theta_{i}}\right|\overset{P}{⟶}0,\;\;\;n^{-1}\sum_{t=1}^{n}\left|\frac{\partial^{2}l_{t}\left(\theta_{0}\right)}{\partial \theta_{i}\partial \theta_{j}}-\frac{\partial^{2}{\tilde{l}}_{t}\left(\theta_{0}\right)}{\partial \theta_{i}\partial \theta_{j}}\right|\overset{P}{⟶}0.$$

Notice that ${\tilde{\mu }}_{t}\left(\theta \right)$ and ${\tilde{\sigma }}_{t}^{2}\left(\theta \right)$ are stationary approximations of $\mu_{t}\left(\theta \right)$ and $\sigma_{t}^{2}\left(\theta \right)$, since $X_{t}$ is stationary and ergodic, using arguments similar to Proposition 2.1.1 in Straumann (2005) [23], for fixed θ∈Θ, ${\tilde{\mu }}_{t}\left(\theta \right)$ and ${\tilde{\sigma }}_{t}^{2}\left(\theta \right)$, $\mu_{t}\left(\theta \right)$ and $\sigma_{t}^{2}\left(\theta \right)$ are also stationary and ergodic. Hence, similar to the proof of Lemma A2 in Hu and Andrews (2021) [24], it is easy to have

$$n^{-1/2}\sum_{t=1}^{n}\left|\frac{\partial l_{t}\left(\theta_{0}\right)}{\partial \theta_{i}}-\frac{\partial {\tilde{l}}_{t}\left(\theta_{0}\right)}{\partial \theta_{i}}\right|\overset{P}{⟶}0,\;\;\;n^{-1}\sum_{t=1}^{n}\left|\frac{\partial^{2}l_{t}\left(\theta_{0}\right)}{\partial \theta_{i}\partial \theta_{j}}-\frac{\partial^{2}{\tilde{l}}_{t}\left(\theta_{0}\right)}{\partial \theta_{i}\partial \theta_{j}}\right|\overset{P}{⟶}0.$$

Therefore, it suffices to show that

$$\frac{1}{\sqrt{n}}\sum_{t=1}^{n}\frac{\partial l_{t}\left(\theta_{0}\right)}{\partial \theta }\overset{d}{⟶}N\left(0,\Sigma \right),\;\;\;\frac{1}{n}\sum_{t=1}^{n}\frac{\partial^{2}l_{t}\left(\theta_{0}\right)}{\partial \theta \partial \theta^{T}}\overset{P}{⟶}-\Sigma .$$

First, we should guarantee that

(A1) $$\begin{array}{c} E_{\theta_{0}}∥\frac{\partial l_{t}\left(\theta_{0}\right)}{\partial \theta }\frac{\partial l_{t}\left(\theta_{0}\right)}{\partial \theta^{T}}∥<\infty ,\;\;\;E_{\theta_{0}}∥\frac{\partial^{2}l_{t}\left(\theta_{0}\right)}{\partial \theta \partial \theta^{T}}∥<\infty . \end{array}$$

Now we prove the first part of (A1).

$$\begin{array}{cc} E_{\theta_{0}}{\left(\frac{\partial l_{t}\left(\theta_{0}\right)}{\partial \omega }\right)}^{2} & =E_{\theta_{0}}\left[\frac{1}{2\sigma_{t}^{4}\left(\theta_{0}\right)}{\left(\frac{\partial \sigma_{t}^{2}\left(\theta_{0}\right)}{\partial \omega }\right)}^{2}+\frac{1}{\sigma_{t}^{2}\left(\theta_{0}\right)}{\left(\frac{\partial \mu_{t}\left(\theta_{0}\right)}{\partial \omega }\right)}^{2}\right]<\infty . \end{array}$$

Similarly, we can prove other terms, thus, the first part of (A1) holds. The proof of the second part of (A1) is similar, here we omit the details. Under (A1), $\left\{\frac{\partial l_{t}\left(\theta_{0}\right)}{\partial \theta }\right\}$ is a martingale difference sequence with respect to $\left\{F_{t}\right\}$, it follows that at $\theta =\theta_{0}$, $E_{\theta_{0}}\left(\frac{\partial l_{t}\left(\theta_{0}\right)}{\partial \theta }|F_{t-1}\right)=0$, so $E_{\theta_{0}}\left(\frac{\partial l_{t}\left(\theta_{0}\right)}{\partial \theta }\right)=0$. Moreover, we have shown that $\Sigma =E_{\theta_{0}}\left(\frac{\partial l_{t}\left(\theta_{0}\right)}{\partial \theta }\frac{\partial l_{t}\left(\theta_{0}\right)}{\partial \theta^{T}}\right)$ in Section 3.2. Hence $\frac{1}{\sqrt{n}}\sum_{t=1}^{n}\frac{\partial {\tilde{l}}_{t}\left(\theta_{0}\right)}{\partial \theta }\overset{d}{⟶}N\left(0,\Sigma \right)$ holds by the central limit theorem for martingale difference sequence in Billingsley (1961). Similarly, we have $E_{\theta_{0}}\left(\frac{\partial l_{t}^{2}\left(\theta_{0}\right)}{\partial \theta \partial \theta^{T}}\right)=-\Sigma$. Under Assumption 1, $\frac{1}{n}\sum_{t=1}^{n}\frac{\partial^{2}{\tilde{l}}_{t}\left(\theta_{0}\right)}{\partial \theta_{i}\partial \theta_{j}}\overset{P}{⟶}-\Sigma$ follows from the ergodic theorem. Thus, Lemma A1 is proved. □

Before showing Lemma A2, we have

$${\tilde{T}}_{n}\left(u\right)\equiv {\tilde{l}}_{n}\left(\theta_{0}+\frac{u}{\sqrt{n}}\right)-{\tilde{l}}_{n}\left(\theta_{0}\right),\;u\in R^{q+1},$$

we use ${\tilde{T}}_{n}$ to derive the asymptotic distribution of ${\hat{\theta }}_{n}$.

For any $u\in R^{q+1}$, the Taylor series expansion of ${\tilde{T}}_{n}\left(u\right)$ at $\theta_{0}$ is

(A2) $${\tilde{T}}_{n}\left(u\right)=\frac{1}{\sqrt{n}}\sum_{t=1}^{n}u^{T}\frac{\partial {\tilde{l}}_{t}\left(\theta_{0}\right)}{\partial \theta }+\frac{1}{2n}\sum_{t=1}^{n}u^{T}\frac{\partial^{2}{\tilde{l}}_{t}\left(\theta_{0}\right)}{\partial \theta \partial \theta^{T}}u+\frac{1}{2n}\sum_{t=1}^{n}u^{T}\left[\frac{\partial^{2}{\tilde{l}}_{t}\left(\theta^{*}\right)}{\partial \theta \partial \theta^{T}}-\frac{\partial^{2}{\tilde{l}}_{t}\left(\theta_{0}\right)}{\partial \theta \partial \theta^{T}}\right]u,$$

where $\theta^{*}=\theta_{n}^{*}\left(u\right)$ is on the line segment connecting $\theta_{0}$ and $\theta_{0}+\frac{u}{\sqrt{n}}$. For Euclidean distance ‖·‖ and any compact set $K\subset R^{q+1}$, $\sup_{u\in K}\left\|\theta^{*}-\theta_{0}\right\|\to 0$, as n→∞.

**Lemma** **A2.**
*Under Assumptions 1 and 2, when n→∞,*

$$\frac{1}{n}\sum_{t=1}^{n}\left[\frac{\partial^{2}{\tilde{l}}_{t}\left(\theta^{*}\right)}{\partial \theta \partial \theta^{T}}-\frac{\partial^{2}{\tilde{l}}_{t}\left(\theta_{0}\right)}{\partial \theta \partial \theta^{T}}\right]\overset{P}{⟶}0.$$

**Proof.** Similar to Lemma A1, for any 1≤i,j≤q+1,

(A3) $$\frac{1}{n}\sum_{t=1}^{n}∥\frac{\partial^{2}l_{t}\left(\theta_{0}\right)}{\partial \theta_{i}\partial \theta_{j}}-\frac{\partial^{2}{\tilde{l}}_{t}\left(\theta_{0}\right)}{\partial \theta_{i}\partial \theta_{j}}∥\overset{P}{⟶}0.$$

Using arguments similar to the proof of Theorem 2.2 of Francq and Zakoïan (2004) [20], it suffices to show

(A4) $$\frac{1}{n}\sum_{t=1}^{n}\left[\frac{\partial^{2}l_{t}\left(\theta^{*}\right)}{\partial \theta_{i}\partial \theta_{j}}-\frac{\partial^{2}l_{t}\left(\theta_{0}\right)}{\partial \theta_{i}\partial \theta_{j}}\right]\overset{P}{⟶}0.$$

By the Taylor series expansion, we have

$$\frac{1}{n}\sum_{t=1}^{n}\frac{\partial^{2}l_{t}\left(\theta^{*}\right)}{\partial \theta_{i}\partial \theta_{j}}=\frac{1}{n}\sum_{t=1}^{n}\frac{\partial^{2}l_{t}\left(\theta_{0}\right)}{\partial \theta_{i}\partial \theta_{j}}+\frac{1}{n}\sum_{t=1}^{n}\frac{\partial }{\partial \theta_{k}}\left(\frac{\partial^{2}l_{t}\left(\theta^{**}\right)}{\partial \theta_{i}\partial \theta_{j}}\right)\left(\theta^{*}-\theta_{0}\right),$$

here $\theta^{**}=\theta_{n}^{**}\left(u\right)$ is on the line segment connecting $\theta_{0}$ and $\theta^{*}$, such that for any *u*, we have $\|\theta^{**}-\theta_{0}\|\to 0$a.s.,n→∞. From (A2), $\|\theta^{*}-\theta_{0}\|\to 0$a.s, so

$$\frac{1}{n}\sum_{t=1}^{n}\frac{\partial }{\partial \theta_{k}}\left(\frac{\partial^{2}l_{t}\left(\theta^{**}\right)}{\partial \theta_{i}\partial \theta_{j}}\right)\left(\theta^{*}-\theta_{0}\right)\to 0,\;a.s.$$

if

(A5) $$\underset{n\to \infty }{lim sup}∥\frac{1}{n}\sum_{t=1}^{n}\frac{\partial }{\partial \theta_{k}}\left(\frac{\partial^{2}l_{t}\left(\theta^{**}\right)}{\partial \theta_{i}\partial \theta_{j}}\right)∥<\infty ,\;a.s.$$

Then we have

$$\frac{1}{n}\sum_{t=1}^{n}\frac{\partial^{2}l_{t}\left(\theta^{*}\right)}{\partial \theta_{i}\partial \theta_{j}}\to \frac{1}{n}\sum_{t=1}^{n}\frac{\partial^{2}l_{t}\left(\theta_{0}\right)}{\partial \theta_{i}\partial \theta_{j}}\;a.s.,$$

so (A4) is proved. Using arguments similar to the proof of Theorem 2.2 of Francq and Zakoïan (2004) [20], there exists a neighborhood $\nu (\theta_{0})$, that

(A6) $$E_{\theta_{0}}\underset{\theta \in \nu (\theta_{0})\cap \Theta }{\sup}∥\frac{\partial }{\partial \theta_{k}}\left(\frac{\partial^{2}l_{t}\left(\theta \right)}{\partial \theta_{i}\partial \theta_{j}}\right)∥<\infty ,\;\;\;\underset{\theta \in \nu (\theta_{0})}{\sup}∥\frac{1}{n}\sum_{t=1}^{n}\left[\frac{\partial^{2}l_{t}\left(\theta \right)}{\partial \theta_{i}\partial \theta_{j}}-\frac{\partial^{2}{\tilde{l}}_{t}\left(\theta \right)}{\partial \theta_{i}\partial \theta_{j}}\right]∥\overset{P}{⟶}0.$$

Therefore, by the ergodic theorem, we have

$$\begin{array}{cc} \underset{n\to \infty }{lim sup}∥\frac{1}{n}\sum_{t=1}^{n}\frac{\partial }{\partial \theta_{k}}\left(\frac{\partial^{2}l_{t}\left(\theta^{**}\right)}{\partial \theta_{i}\partial \theta_{j}}\right)∥\; & \leq \underset{n\to \infty }{lim sup}\frac{1}{n}\sum_{t=1}^{n}\underset{\theta \in \nu (\theta_{0})\cap \Theta }{\sup}∥\frac{\partial }{\partial \theta_{k}}\left(\frac{\partial^{2}l_{t}\left(\theta \right)}{\partial \theta_{i}\partial \theta_{j}}\right)∥\\ \; & =E_{\theta_{0}}\underset{\theta \in \nu (\theta_{0})\cap \Theta }{\sup}∥\frac{\partial }{\partial \theta_{k}}\left(\frac{\partial^{2}l_{t}\left(\theta \right)}{\partial \theta_{i}\partial \theta_{j}}\right)∥<\infty , \end{array}$$

so (A5) is proved. In view of (A3), (A4) and (A6), we obtain Lemma A2. □

**Lemma** **A3.**
*For any compact set $K\in R^{q+1}$ and any ε>0,*

$$\lim_{\sigma \to 0}\underset{n\to \infty }{lim sup}P\left(\underset{u,v\in K,\|u-v\|<\sigma }{\sup}\left|{\tilde{T}}_{n}\left(u\right)-{\tilde{T}}_{n}\left(v\right)\right|\geq \varepsilon \right)=0.$$

**Proof.** For any ϵ>0, by (A2) we have

$$\begin{array}{cc} \; & \lim_{\delta \to 0}\underset{n\to \infty }{lim sup}P\left(\underset{u,v\in K,\|u-v\|<\delta }{\sup}\left|{\tilde{T}}_{n}\left(u\right)-{\tilde{T}}_{n}\left(v\right)\right|\geq \varepsilon \right)\\ \; & \leq \lim_{\delta \to 0}\underset{n\to \infty }{lim sup}P\left(\underset{u,v\in K,\|u-v\|<\delta }{\sup}\left|\frac{1}{\sqrt{n}}\sum_{t=1}^{n}{\left(u-v\right)}^{T}\frac{\partial {\tilde{l}}_{t}\left(\theta_{0}\right)}{\partial \theta }\right|\geq \frac{\epsilon }{3}\right)\\ \; & +\lim_{\delta \to 0}\underset{n\to \infty }{lim sup}P\left(\underset{u,v\in K,\|u-v\|<\delta }{\sup}\left|\frac{1}{n}\left(\sum_{t=1}^{n}u^{T}\frac{\partial^{2}{\tilde{l}}_{t}\left(\theta_{0}\right)}{\partial \theta \partial \theta^{T}}u-\sum_{t=1}^{n}v^{T}\frac{\partial^{2}{\tilde{l}}_{t}\left(\theta_{0}\right)}{\partial \theta \partial \theta^{T}}v\right)\right|\geq \frac{2\epsilon }{3}\right)\\ \; & +\lim_{\delta \to 0}\underset{n\to \infty }{lim sup}P\left\{\underset{u,v\in K,\|u-v\|<\delta }{\sup}\left|\frac{1}{n}\left[\sum_{t=1}^{n}u^{T}\left(\frac{\partial^{2}{\tilde{l}}_{t}\left(\theta^{*}\right)}{\partial \theta \partial \theta^{T}}-\frac{\partial^{2}{\tilde{l}}_{t}\left(\theta_{0}\right)}{\partial \theta \partial \theta^{T}}\right)u\right.\right.\right.\\ \; & \left.\left.\left.-\sum_{t=1}^{n}v^{T}\left(\frac{\partial^{2}{\tilde{l}}_{t}\left(\theta^{*}\right)}{\partial \theta \partial \theta^{T}}-\frac{\partial^{2}{\tilde{l}}_{t}\left(\theta_{0}\right)}{\partial \theta \partial \theta^{T}}\right)v\right]\right|\geq \frac{2\epsilon }{3}\right\}. \end{array}$$

Because of Lemmas A1 and A2, we have

$$\frac{1}{\sqrt{n}}\sum_{t=1}^{n}\frac{\partial {\tilde{l}}_{t}\left(\theta_{0}\right)}{\partial \theta }=O_{p}\left(1\right),\;\;\;\frac{1}{n}\sum_{t=1}^{n}\frac{\partial^{2}{\tilde{l}}_{t}\left(\theta_{0}\right)}{\partial \theta \partial \theta^{T}}=O_{p}\left(1\right),$$

$$\frac{1}{n}\sum_{t=1}^{n}\left[\frac{\partial^{2}{\tilde{l}}_{t}\left(\theta^{*}\right)}{\partial \theta \partial \theta^{T}}-\frac{\partial^{2}{\tilde{l}}_{t}\left(\theta_{0}\right)}{\partial \theta \partial \theta^{T}}\right]=o_{p}\left(1\right),$$

where $O_{p}\left(1\right)$ and $o_{p}\left(1\right)$ for vector and matrix means $O_{p}\left(1\right)$ and $o_{p}\left(1\right)$ for every elements. By the compactness of K, we have

$$\lim_{\delta \to 0}\underset{n\to \infty }{lim sup}P\left(\underset{u,v\in K,\|u-v\|<\delta }{\sup}\left|\frac{1}{\sqrt{n}}\sum_{t=1}^{n}{\left(u-v\right)}^{T}\frac{\partial {\tilde{l}}_{t}\left(\theta_{0}\right)}{\partial \theta }\right|\geq \frac{\epsilon }{3}\right)=0,$$

$$\lim_{\delta \to 0}\underset{n\to \infty }{lim sup}P\left(\underset{u,v\in K,\|u-v\|<\delta }{\sup}\left|\frac{1}{n}\left(\sum_{t=1}^{n}u^{T}\frac{\partial^{2}{\tilde{l}}_{t}\left(\theta_{0}\right)}{\partial \theta \partial \theta^{T}}u-\sum_{t=1}^{n}v^{T}\frac{\partial^{2}{\tilde{l}}_{t}\left(\theta_{0}\right)}{\partial \theta \partial \theta^{T}}v\right)\right|\geq \frac{2\epsilon }{3}\right)=0,$$

$$\begin{array}{cc} \lim_{\delta \to 0}\underset{n\to \infty }{lim sup}P & \left\{\underset{u,v\in K,\|u-v\|<\delta }{\sup}\left|\frac{1}{n}\left[\sum_{t=1}^{n}u^{T}\left(\frac{\partial^{2}{\tilde{l}}_{t}\left(\theta^{*}\right)}{\partial \theta \partial \theta^{T}}-\frac{\partial^{2}{\tilde{l}}_{t}\left(\theta_{0}\right)}{\partial \theta \partial \theta^{T}}\right)u\right.\right.\right.\\ \; & \left.\left.\left.-\sum_{t=1}^{n}v^{T}\left(\frac{\partial^{2}{\tilde{l}}_{t}\left(\theta^{*}\right)}{\partial \theta \partial \theta^{T}}-\frac{\partial^{2}{\tilde{l}}_{t}\left(\theta_{0}\right)}{\partial \theta \partial \theta^{T}}\right)v\right]\right|\geq \frac{2\epsilon }{3}\right\}=0, \end{array}$$

which completes our proof. □

### Appendix A.6. Proof of Theorem 2

**Proof.** Let $T\left(u\right)=u^{T}N\left(0,\Sigma \right)-\frac{1}{2}u^{T}\Sigma u$, where *N* is a multivariate Gaussian random vector with mean 0 and covariance matrix Σ. By Lemmas A1 and A2, for any $u\in R^{q+1}$ and n→∞, the finite dimensional distributions of ${\tilde{T}}_{n}$ converge to those of *T*: ${\tilde{T}}_{n}\left(u\right)\to T\left(u\right)$. By Lemma A3, similar to Hu (2016) [9], ${\tilde{T}}_{n}\left(u\right)$ is tight on the continuous function space C(K) for any compact set $K\in R^{q+1}$. So by Theorem 7.1 in Billingsley (1999) [25], ${\tilde{T}}_{n}\left(\cdot \right)\to T\left(\cdot \right)$ on C(K). From Appendix A.4 and Lemma A1, Σ is positive finite and invertible, meanwhile, T(·) is concave with the unique maximum $\Sigma^{-1}N\left(0,\Sigma \right)=N\left(0,\Sigma^{-1}\right)$. ${\tilde{T}}_{n}\left(\cdot \right)$ is maximized at $u_{\max}=\sqrt{n}\left({\hat{\theta }}_{n}-\theta_{0}\right)$. Thus, the result of Theorem 2 can be proved by the proof of Lemma 2.2 and Remark 1 in Davis et al. (1992) [26]. □
